# The Ages and Stages Questionnaire and Neurodevelopmental Impairment in Two-Year-Old Preterm-Born Children

**DOI:** 10.1371/journal.pone.0133087

**Published:** 2015-07-20

**Authors:** Jorien M. Kerstjens, Ard Nijhuis, Christian V. Hulzebos, Deirdre E. van Imhoff, Aleid G. van Wassenaer-Leemhuis, Ingrid C. van Haastert, Enrico Lopriore, Titia Katgert, Renate M. Swarte, Richard A. van Lingen, Twan L. Mulder, Céleste R. Laarman, Katerina Steiner, Peter H. Dijk

**Affiliations:** 1 Division of Neonatology, Department of Pediatrics, Beatrix Children’s Hospital, University Medical Center Groningen, Groningen, The Netherlands; 2 Department of Neonatology, Emma Children's Hospital Academic Medical Center, Amsterdam, The Netherlands; 3 Department of Neonatology, Wilhelmina Children's Hospital/University Medical Center Utrecht, Utrecht, The Netherlands; 4 Division of Neonatology, Department of Pediatrics, Leiden University Medical Center, Leiden, The Netherlands; 5 Department of Medical Psychology, Máxima Medical Center, Veldhoven, The Netherlands; 6 Department of Neonatology, Erasmus MC-Sophia, Rotterdam, The Netherlands; 7 Princess Amalia Department of Pediatrics, Department of Neonatology, Isala, Zwolle, The Netherlands; 8 Department of Pediatrics, Maastricht University Medical Center, GROW–School for Oncology and Developmental Biology, Maastricht, The Netherlands; 9 Division of Neonatology, Department of Pediatrics, VU University Medical Center, Amsterdam, The Netherlands; 10 Division of Neonatology, Department of Pediatrics, Radboud University Medical Centre Nijmegen, Nijmegen, The Netherlands; Centre Hospitalier Universitaire Vaudois, FRANCE

## Abstract

**Objective:**

To test the ability of the Ages and Stages Questionnaire, Third Edition (ASQ3) to help identify or exclude neurodevelopmental impairment (NDI) in very preterm-born children at the corrected age of two.

**Methods:**

We studied the test results of 224 children, born at <32 postmenstrual weeks, who had scores on ASQ3 and Bayley Scales of Infant and Toddler Development, Third Edition (BSIDIII) and neurological examination at 22–26 months’ corrected age. We defined NDI as a score of <70 on the cognitive—or motor composite scale of BSIDIII, or impairment on neurological examination or audiovisual screening. We compared NDI with abnormal ASQ3 scores, i.e., < -2SDs on any domain, and with ASQ3 total scores. To correct for possible overestimation of BSIDIII, we also analyzed the adjusted BSIDIII thresholds for NDI, i.e., scores <80 and <85.

**Results:**

We found 61 (27%) children with abnormal ASQ3 scores, and 10 (4.5%) children who had NDI with original BSIDIII thresholds (<70). Twelve children had NDI at BSIDIII thresholds at <80, and 15 had <85. None of the 163 (73%) children who passed ASQ3 had NDI. The sensitivity of ASQ3 to detect NDI was excellent (100%), its specificity was acceptable (76%), and its negative predictive value (NPV) was 100%. Sensitivity and NPV remained high with the adjusted BSIDIII thresholds.

**Conclusion:**

The Ages and Stages Questionnaire is a simple, valid and cost-effective screening tool to help identify and exclude NDI in very preterm-born children at the corrected age of two years.

## Introduction

Identifying neurodevelopmental impairment (NDI) at a young age is important for children at risk for developmental problems, brought on by preterm birth or neonatal encephalopathy. Early recognition of NDI is one of the key recommendations of the American Academy of Pediatrics [1]. It improves the chances of early intervention within the windows of opportunity for development [2,3].

The standardized tool used most frequently to assess development at a young age is the Bayley Scales of Infant and Toddler Development (BSID). This tool is often referred to as the gold standard for infant development and is used in structured follow-up care of and in research on very preterm-born children and other high risk groups [4]. A practical disadvantage, however, is that it is laborious and costly. It takes approximately 50 to 90 minutes to complete and it must be administered by trained professionals. Furthermore, it requires a fully cooperative child who has sufficient neurosensory skills [5].

Neurodevelopmental outcome is usually expressed as rates of NDI, whereby sensory impairment and neurological dysfunction leading to cerebral palsy are scored independently and added to BSID scores below -2SD [6,7].

If the purpose of neurodevelopmental testing is to identify or exclude NDI, then using professional-completed or parent-completed developmental screeners may be less time consuming and more cost-effective [8]. Currently, the most widely used parent-completed questionnaires for young children is the Ages and Stages Questionnaire (ASQ) [9]. ASQ has shown high sensitivity and specificity for detecting developmental delay [10–13]. Earlier, the ASQ, Second Edition (ASQ2) had been compared to BSID Second Edition (BSIDII) with promising results [10–12]. Recently, the ASQ, Third Edition (ASQ3) was compared with BSIDIII in a mixed term-born and preterm-born population of children aged 8, 18, and 30 months, yielding adequate sensitivity and specificity [13]. To our knowledge, the ASQ3-24 month’s version has not been compared to BSIDIII or to formal NDI assessments at 24 months’ corrected age, even though NDI at 24 months is an important end-point in many clinical studies.

The objective of our study was to test the ability of the ASQ3 24 months’ version to correctly identify or exclude NDI in very preterm-born children at the age of two.

## Methods

### Population and participants

Participants were two-year-old children who were born preterm at < 32 weeks’ postmenstrual age, without major congenital abnormalities, and who had been treated in one of the ten tertiary Neonatal Intensive Care Units (NICU’s) in the Netherlands. These children were initially recruited as part of the BARTrial (ISRCTN74465643), a prospective, randomized controlled, multicenter study that aimed to reduce bilirubin induced neurological dysfunction [14–15]. A total of 301 survivors for whom we had results on BSIDIII and the complete neurological and sensory examinations, and whose parents had completed the ASQ3 within a time window of 2 months (22–26 months), were included in this part of the study. The Medical Ethics Review Board of University Medical Center Groningen approved the study. Written informed consent was obtained from the parents or legal guardian of each participating infant. This study was conducted according to the principles expressed in the Declaration of Helsinki.

### Measures

The Ages and Stages Questionnaire (ASQ) is a parent-completed screening tool [9]. It measures developmental milestones in five domains (communication, fine motor, gross motor, problem solving ability, and personal-social functioning). Each domain consists of six questions. Parents indicate whether their child has mastered the milestone (yes, 10 points), partly/inconsistently (partly, 5 points), or not yet (no, 0 points). The ASQ2 was translated into Dutch (Dutch-ASQ) and back-translated once prior to the study to check for problems relating to the translation [16]. Pending the Dutch ASQ norms, we used the original United States (U.S.) ASQ3 cut-off norms that had been generated from a large standardized sample of American children. We chose to use the ASQ3 norms for the Dutch-ASQ because to all intents and purposes the domain items of the original English ASQ2 and ASQ3 24 months versions are the same except for some minor alterations in the English wording of the questions. We defined “failing ASQ3”, which indicates possible developmental delay, as a score of more than 2 SDs below the mean score for the U.S. reference group (ASQ3) on any of the five domains, or a domain with an invalid score due to more than two missing answers on that domain, both in accordance with the manual [9].

The Bayley Scales of Infant and Toddler Development, Third Edition (BSIDIII), is a widely used tool to assess development of infants and toddlers from 0.5 to 42.5 months of age [4,5]. It quantifies cognitive, language, and motor skills through a series of standardized test items and social and adaptive skills through parental questionnaires. In 2000, the normative data of BSIDIII were generated from a standardization sample of more than 1400 American children aged 1 to 42 months [5]. BSIDIII provides five norm-referenced composite scores (cognitive, language, motor, social and adaptive) with a mean of 100 and a SD of ±15. The original U.S. cut-off scores for delay on BSIDIII were set at -2SDs (i.e at 70) in accordance with the original manual. In this study we assessed the cognitive and composite motor score of BSIDIII, in analogy to its two predecessors of BSIDII: the mental development index (MDI) and the psychomotor development index (PDI) [17]. Due to increasing concerns about possible overestimation of BSIDIII cognitive and motor scales in comparison to BSIDII [18–20], we also examined additional cut-off composite scores for the BSIDIII <80 and <85, hereafter referred to as “adjusted” BSIDIII thresholds, in accordance with current recommendations [19–20].

We defined neurodevelopmental impairment (NDI) as either a BSIDIII cognitive score or composite motor score of < 70, moderate or severe cerebral palsy according to neurological examination [21], or a score > II on the Gross Motor Function Classification System (GMFCS) [22], bilateral blindness (visual acuity of <20/200), or bilateral deafness (bilateral hearing loss of >40 dB). For the “adjusted” NDI assessments, cut-off scores for BSIDIII were raised to <80 and <85, respectively, whereby the other outcome measures remained unchanged.

### Procedures

Parents received the Dutch-ASQ at home together with an invitation for this part of the study a few weeks before to the planned follow-up visit. During this visit a trained professional administered the BSIDIII cognitive and motor scales, conducted an age appropriate examination, including a neurological examination according to Touwen [23], and did visual and hearing assessments. In addition, we collected medical data concerning gestational age, small for gestational age (SGA) status (<P10) [24] and information about medical complications before discharge.

The Dutch-ASQ forms were collected during the visit. If parents had not yet completed the questionnaire, they were reminded once or twice by telephone afterwards. The professionals who administered BSIDIII were blind as to the ASQ results. A time window for the Dutch-ASQ was set at two months around the second birthday (22 to 26 months) corrected for gestational age in accordance with the guidelines in the most recent ASQ manual [9]. BSIDIII results were analyzed for all children with BSIDIII results within the range of 21 to 30 months’ corrected age.

### Statistical analyses

We calculated percentages for children with abnormal ASQ scores for different demographic variables and biological risk factors. The differences in percentages of abnormal ASQ results for children with and without risk factors were tested with chi-square analyses. We cross-tabulated the results on the ASQ3 and BSIDIII and calculated sensitivity, specificity, positive predictive value (PPV) and negative predictive values (NPV) for NDI at original and adjusted thresholds. Furthermore we added up all ASQ domain scores to form an ASQ total score and compared this to the additive BSID cognitive and composite motor scores using linear regression analysis and receiver operator characteristics. Incomplete BSID scores due to CP or severe mental impairment were imputed at 50 and incomplete ASQ scores were imputed with 0 points per item without a valid score. We investigated on which ASQ3 domains the very-preterm children failed most often, both overall as well as for children with and without NDI. Statistical analyses were performed with SPSS version 19 (SPSS Inc., Chicago, Il, USA). Statistical significance was preset at *P*< 0.05.

## Results

**Fig 1:**
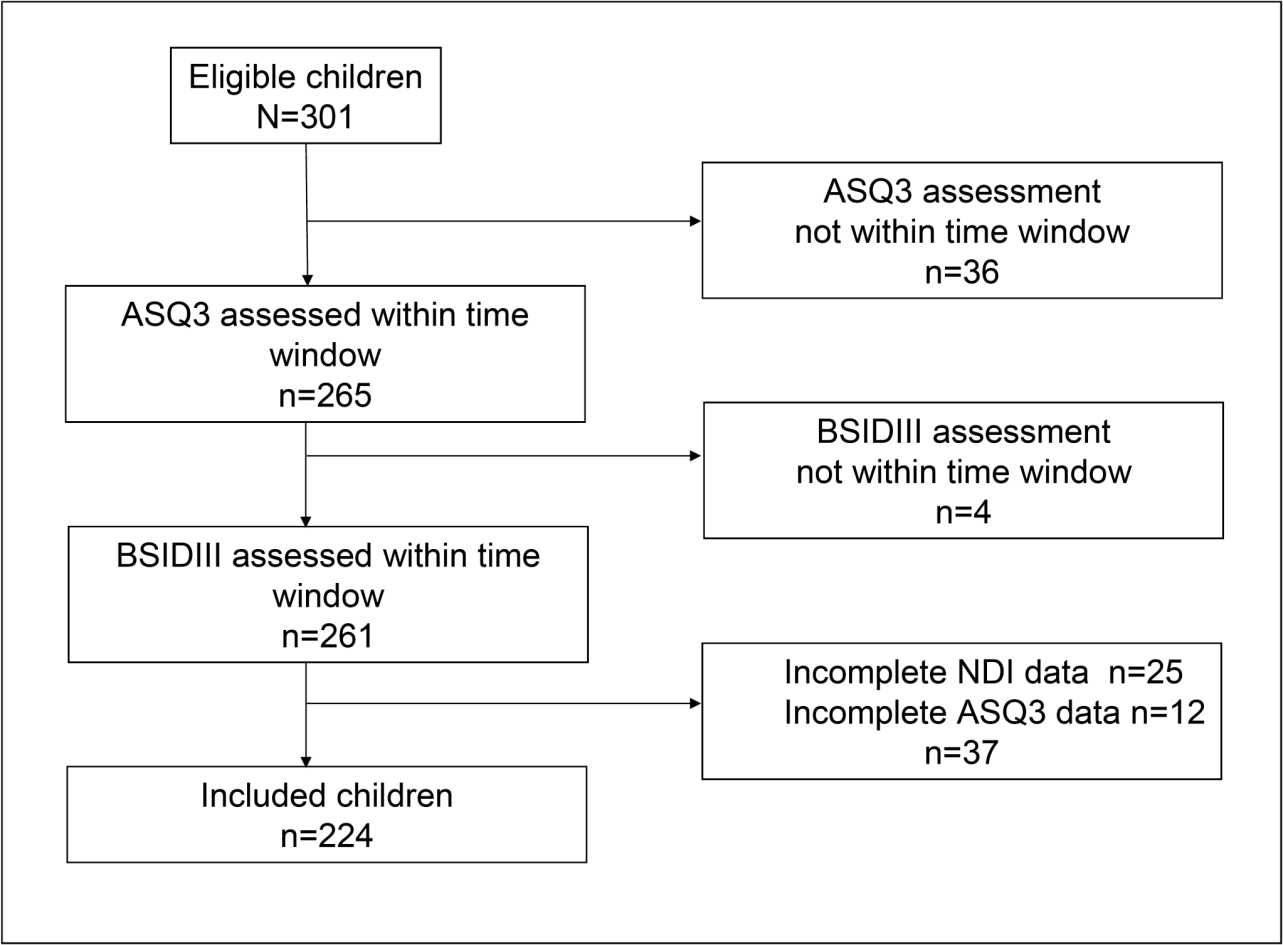
Flowchart with numbers of eligible and included children. ASQ3; Ages and Stages Questionnaire, Third Edition, time window 22–26 months corrected age. BSIDIII: Bayley Scales of Infant and Toddler Development, Third Edition, time window 21–30 months corrected age. NDI: Neurodevelopmental Impairment: BSIDIII cognitive score or composite motor score <70, bilateral blindness/deafness or cerebral palsy.

Out of a total of 301 eligible children, 261 (87%) had results within the time windows of ASQ3 and BSIDIII. Data on 37 children were incomplete so that we were unable to determine NDI and ASQ scores in 25 and 12 cases, respectively. Eventually, we compared ASQ3 scores and NDI results of 224 (74%) out of 301 children (Fig 1). The characteristics of these children are described in Table 1 **.** Children who were excluded did not differ significantly from the other participants on demographic characteristics, BSIDIII results, or rates of NDI (S1 Table). The mean age at assessment was 24 months for both ASQ3 and BSIDIII.

**Table 1:** Characteristics of the study sample.

Characteristic	N = 224
**Gestational age in weeks**	29.3 ± 1.9 [24–31]
**Birth weight in grams**	1270 ± 337 [600–2300]
**Age of assessment ASQ3 in months**	24 ± 0.98 [22–26]
**Age of assessment BSIDIII in months**	24 ± 1.19 [22–30]
**Total ASQ3 score**	250 (225–270)
**BSIDIII cognitive score**	100 (95–110)
**BSIDIII composite motor score**	100 (94–110)

Data are expressed as mean ± SD and [range], or median and (25–75 percentile)

ASQ3: Ages and Stages Questionnaire, Third Edition

BSIDIII: Bayley Scales of Infant and Toddler Development, Third Edition

Out of 224 children 61(27%) had abnormal ASQ3 scores and 10 (4.5%) had NDI, using original cut-off scores for BSIDIII at -2SDs (<70) (Table 2). Significantly more children with a gestational age of <28 weeks failed ASQ3. Children who were part of a multiple birth, or who had intraventricular hemorrhage (IVH) or periventricular leukomalacia (PVL) >grade 2, or bronchopulmonary dysplasia (BPD), also failed ASQ3 more often.

**Table 2:** Characteristics of children who failed the ASQ3 or had NDI.

		All	Failure ASQ3		NDI	
		N = 224	n = 61		n = 10	
Characteristic		*n*	*n (%)*	*P*	*n (%)*	*P*
**Gestational age**	< 28 weeks	61	25 (41)	**0.005**	4 (7)	0.353
	>28weeks	163	36 (22)		6 (4)	
**SGA** ^1^	<P10	45	12 (27)	0.924	1 (2)	0.415
	>P10	179	49 (27)		9 (5)	
**Gender**	Male	122	37 (30)	0.255	7 (6)	0.313
	Female	102	24 (24)		3 (3)	
**Multiple birth**	Multiple	71	27 (38)	**0.013**	5 (7)	0.203
	Single	153	34 (22)		5 (3)	
**Ethnicity mother** *n = 221*	Caucasian	206	57 (28)	0.933	10 (5)	0.382
	Non-Caus.	15	4 (27)		0 (0)	
**Education mother** ^2^ *n = 208*	Low	28	7 (25)	0.670	0 (0)	0.226
	Middle/high	180	52 (29)		9 (5)	
**NEC**	Yes	9	3 (33)	0.675	1 (11)	0.324
	No	215	58 (27)		9 (4)	
**IVH or PVL** ^3^	Yes	13	8 (62)	**0.004**	0 (0)	0.422
	No	211	53 (23)		10 (5)	
**BPD** ^4^	Yes	24	11 (46)	**0.030**	1 (4)	0.940
	No	200	50 (25)		9 (5)	
**Sepsis** ^5^	Yes	5	3 (60)	0.096	1 (20)	0.089
	No	219	58 (27)		9 (4)	

Data are presented as numbers (N, n), percentages (%) of children with abnormal ASQ3 scores and NDI and P-values in chi-square analyses. N = 224 unless stated otherwise, ASQ3: Ages and Stages Questionnaire, Third Edition, BSIDIII: Bayley Scales of Infant and Toddler Development, Third Edition.NDI: neurodevelopmental impairment: BSIDIII cognitive score or composite motor score of <70, bilateral blindness/deafness or cerebral palsy. Failure ASQ3: a score of >2 SD below the mean score for the US reference group on any domain.

^1^ SGA: small for gestational age, birth weight < P10 on Dutch reference growth chart.

^2^ low education of mother is less than 5 years high school education.

NEC is necrotizing enterocolitis

^3^ IVH or PVL: intraventricular hemorrhage ≥ grade 3 (Papile) or periventricular leukomalacia ≥ grade 3. (De Vries)

^4^ BPD: bronchopulmonary dysplasia defined as oxygen dependence at 36 weeks’ gestation.

^5^ Sepsis: clinical signs of septicemia and positive blood culture.

There were 10 (4.5%) children with NDI at the original BSIDIII cut-off thresholds of <70, 12 (5.4%) with an adjusted threshold of <80, and 15 (7.0%) with an adjusted threshold of <85 (Table 3 and S2 Table). Sensitivity at the original thresholds for the BSIDIII to detect NDI was 100% and specificity 76%. The negative predictive value (NPV) was 100% and positive predictive value (PPV) 16%. With adjusted composite score thresholds at <80 and <85 for BSIDIII, ASQ3 correctly identified 12 out of 12 and 13 out of 15 children with NDI; sensitivity and NPV remained high. These figures were not essentially different in the subgroup of children born before 28 weeks of gestation (S3 Table).

**Table 3:** ASQ3 versus NDI with original (<70) and adjusted BSID thresholds (<80 and <85), sensitivity, specificity, negative and positive predictive values.

	NDI with BSIDIII<70		NDI with BSIDIII<80		NDI with BSIDIII<85	
	Yes	No	Yes	No	Yes	No
**Failure ASQ3** (n = 61)	10	51	12	49	13	48
**Normal ASQ3** (n = 163)	0	163	0	163	2	161
**Total** (N = 224)	10	214	12	212	15	209
**Sensitivity**	100% (10/10)		100% (12/12)		87% (13/15)	
**Specificity**	76 (163/214)		77% (163/212)		77% (161/209)	
**Negative Predictive Value**	100% (163/163)		100% (163/163)		99% (161/163)	
**Positive Predictive Value**	16% (10/61)		20% (12/61)		21% (13/61)	

Data are presented as numbers and percentages.

ASQ3: Ages and Stages Questionnaire, Third edition.

Failure ASQ3: a score of >-2 SD below the mean score for the U.S. reference group on any domain

BSIDIII: Bayley Scales of Infant and Toddler Development, Third Edition.

NDI with BSIDIII<70: neurodevelopmental impairment with BSIDIII cognitive score or composite motor score of <70, bilateral blindness/deafness and/or cerebral palsy.

NDI with BSIDIII<80: neurodevelopmental impairment with BSIDIII cognitive score or composite motor score of <80, bilateral blindness/deafness and/or cerebral palsy.

NDI with BSIDIII< 85: neurodevelopmental impairment with BSIDIII cognitive score or composite motor score of <85, bilateral blindness/deafness and/or cerebral palsy.

**Fig 2:**
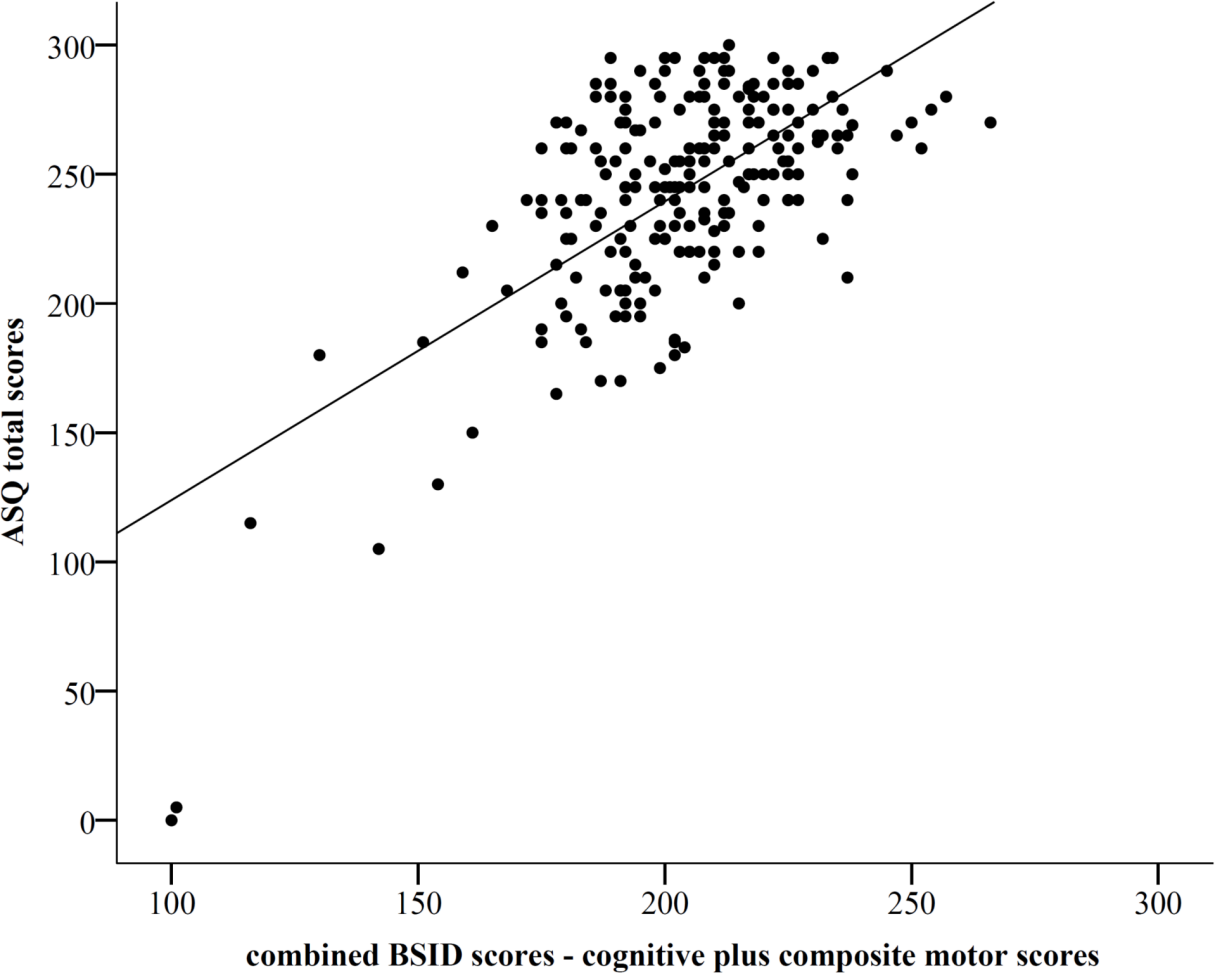
Correlation between BSIDIII combined cognitive and composite motor scores and ASQ3 total scores. R square is 0.417, p<0.001. Incomplete BSID scores due to CP or severe mental impairment were imputed at 50 and incomplete ASQ scores were imputed with 0 points per item without a valid score.

**Fig 3:**
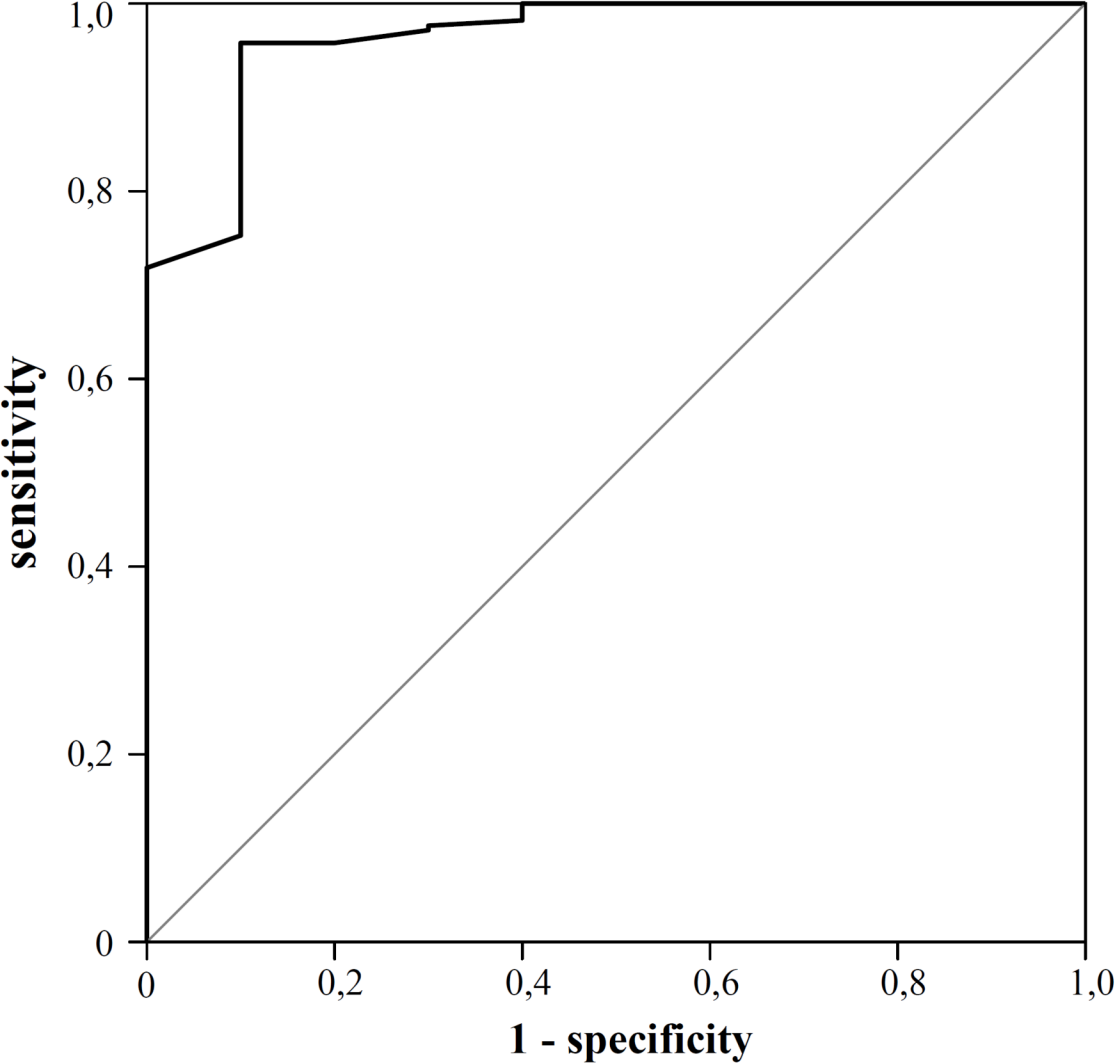
ROC curve for NDI with BSIDIII threshold< 70 versus ASQ3 total scores. Area under the curve 96% p<0.0001

**Fig 4:**
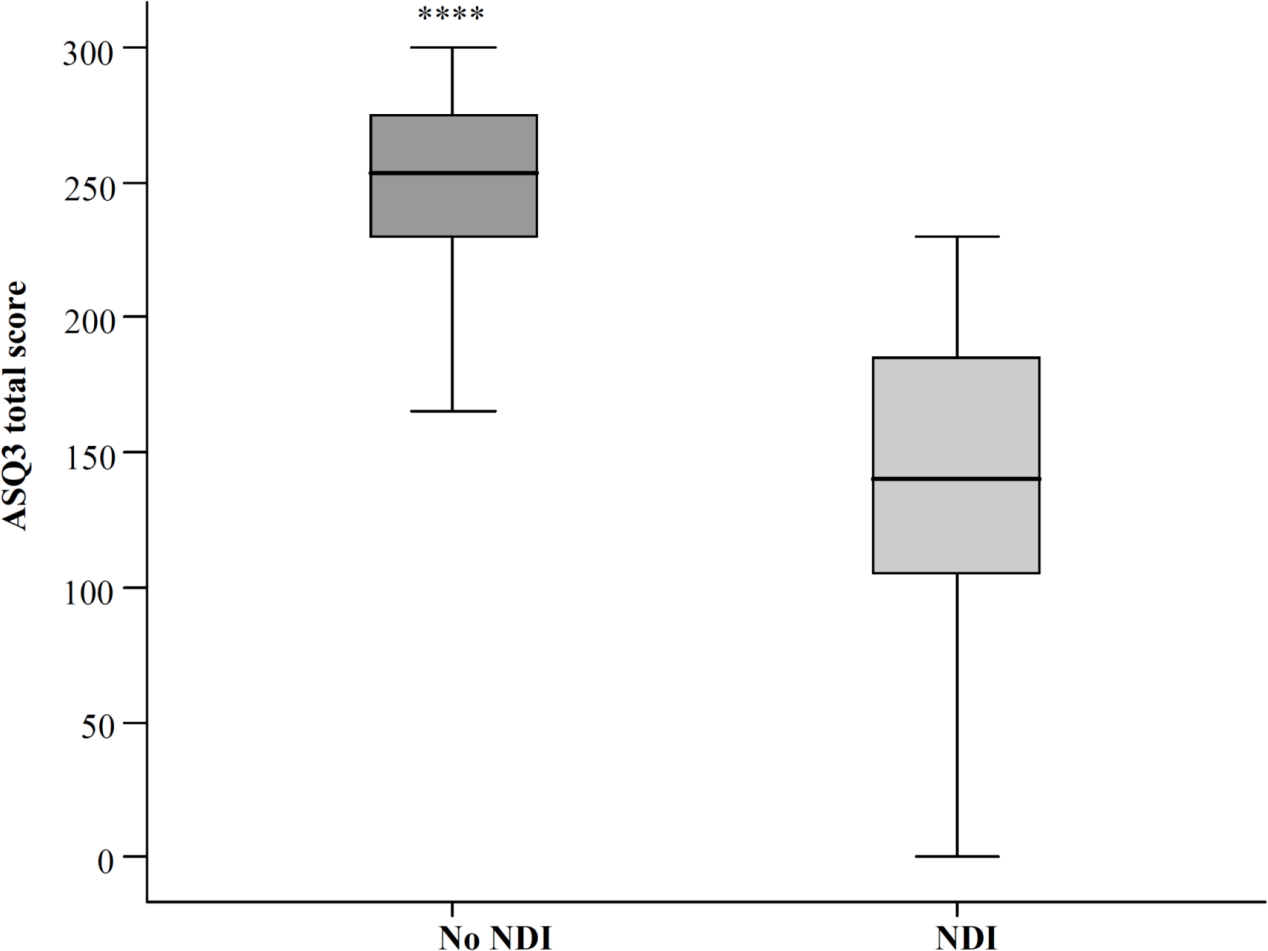
ASQ3 total scores for children with No NDI and children with NDI. NDI with BSID threshold set at < 70. Incomplete BSIDIII scores due to CP or severe mental impairment were imputed at 50 and incomplete ASQ3 scores were imputed with 0 points per item without a valid score.**** *p*< 0.0001

Fig 2 shows the significant correlation between ASQ-total scores and combined BSID scores, with a relatively wide range (R-square is 0.43, p<0.001). A cut-off for the ASQ total score below 188 led to a sensitivity of 96% with a specificity of 90% to detect NDI with a BSIDIII threshold set at < 70, area under the curve in the ROC curve (Fig 3) was 96% (p<0.0001). Fig 4 shows the ranges in total ASQ scores for children with NDI and those without NDI (No-NDI), p<0.001. Children who passed ASQ3 had significantly higher cognitive scores and composite motor scores than children who failed ASQ3 (P<0.001), and who failed the ASQ without NDI (P<0.001) (S4 Table).

More than half, i.e. 34 out of 61 (56%) children with abnormal ASQ3 results, failed on the gross motor domain (Table 4), and 18 (30%) failed on the communication domain; 12 of them (25%) had no NDI (S5 Table). Eighty percent of the children with NDI failed on more than one ASQ3 domain.

**Table 4:** ASQ3 failures and the domains for different NDI thresholds.

	NDI with BSIDIII<70	NDI with BSIDIII<80	NDI with BSIDIII<85	Total
	n = 10	n = 12	n = 15	N = 224
**Failure ASQ3**	10 (100%)	12 (100%)	13 (87%)	61 (27%)
**Domain ASQ3**	**Failure n (%)**	**Failure n (%)**	**Failure n (%)**	**Failure n (%)**
**Communication**	6 (60%)	6 (50%)	6 (40%)	18 (8%)
**Gross Motor**	8 (80%)	9 (75%)	9 (60%)	34 (15%)
**Fine Motor**	3 (30%)	5 (42%)	5 (33%)	12 (5%)
**Problem Solving**	7 (70%)	7 (58%)	7 (47%)	14 (6%)
**Personal Social**	6 (60%)	6 (50%)	7 (47%)	18 (8%)

Data are presented as numbers and percentages.

ASQ3: Ages and Stages Questionnaire, Third edition.

Failure ASQ3: a score of >2 SD below the mean score for the U.S. reference group on any domain

BSIDIII: Bayley Scales of Infant and Toddler Development, Third Edition.

NDI with BSIDIII<70: neurodevelopmental impairment with BSIDIII cognitive score or composite motor score of <70, bilateral blindness/deafness and/or cerebral palsy.

NDI with BSIDIII<80: neurodevelopmental impairment with BSIDIII cognitive score or composite motor score of <80, bilateral blindness/deafness and/or cerebral palsy.

NDI with BSIDIII<85: neurodevelopmental impairment with BSIDIII cognitive score or composite motor score of <85, bilateral blindness/deafness and/or cerebral palsy.

When using ASQ2 thresholds the same 61 children plus an additional number of 23 children had abnormal results: sensitivity remained 100% while specificity dropped to 65%.

## Discussion

This study demonstrated excellent sensitivity and acceptable specificity of ASQ3 to detect NDI in very preterm born children at the corrected age of two years, within the ranges of original and adjusted BSIDIII cut-off scores. The negative predictive value was 100%, which indicates that ASQ3 can be used confidently to screen for NDI. Using ASQ3 and only referring those children with abnormal ASQ results for further testing would reduce the number of children who have to undergo extensive neurodevelopmental testing, thus thoroughly reducing the costs involved in such programs.

Furthermore, the children with ASQ3 failure without NDI had significantly lower BSIDIII scores also, which strengthens the potential use of ASQ as children with borderline BSIDIII scores might benefit from closer follow-up or serial ASQ surveillance.

Our results were in agreement with several other studies that tested the ability of ASQ to detect neurodevelopmental delay despite varying populations, ages of assessment, NDI threshold definitions, and various gold standard tests [10–12,25,26]. The heterogeneity of these studies limits comparability but also supports a broad applicability of ASQ3. Moreover, most of these studies analyzed ASQ2. Our results showed that using ASQ3 thresholds resulted in higher specificity without reducing sensitivity.

To our knowledge, one study compared the new ASQ3 with BSIDIII at 8, 18 and 30 months, with the threshold set at -1SD on at least one domain [13]. It reported an overall sensitivity of 75% and a specificity of 81% in 306 term-born and preterm-born children. The psychometric properties were better for preterm-born children than term-born children and at more advanced test ages. Our study elaborated on these results by adding an important age of assessment, i.e. 24 months, using NDI as outcome measure instead of BSID scores only, and proved ASQ3 thresholds to have better specificity than ASQ2 thresholds. Additionally, we studied a large number of children at one time-point, and we also analyzed two additional thresholds on BSIDIII for NDI. We did so in response to growing concern regarding the differences in test scores of BSIDIII compared to BSIDII [18–20,27,28]. The reasons for the differences, and the question whether the BSIDII underestimates or whether BSIDIII overestimates the outcomes of the children, remain unclear. The BSIDIII administration manual [5] states that both the cognitive score and the composite motor scores are 7 points higher than the MDI and PDI of BSIDII. A possible explanation might be the difference in norm groups, with more mildly impaired children (10%) and proportionally more Hispanic Americans (16%) in the reference group of the BSIDIII in comparison to BSIDII [29]. Several groups found larger mean differences (8 to 15 points), especially in the lower-end scores on several BSIDIII scales compared to BSIDII, with a non-linear correlation [18–20,27,28,31].

We chose <80 and <85 as additional thresholds for the cognitive and composite motor score to define NDI as was suggested by several of these authors [19,20,30,31].

Regardless of exact thresholds on BSIDIII, the question remains whether developmental screening tools can be used reliably to detect which children in high-risk groups should be referred for more in-depth neurodevelopmental assessment. If the aim is to detect NDI, such a screening would reduce workload and would relieve the financial burden of large scale follow-up programs. About three out of four children in this study would have been identified as developing according the norm, without formal developmental testing. Even after applying the adjusted thresholds for the BSIDIII (threshold BSID <85), the workload of testing an additional 163 children to detect two more children with NDI is huge. Depending on the reason for assessment, the available resources, and other possible safety nets to ensure that early identification of children at risk of developmental impairment is guaranteed, this may or may not be considered acceptable. Furthermore, administering serial ASQ’s at different points in time, or administering an ASQ of a more advanced age than the corrected age (e.g. calendar age) of the child in order to improve sensitivity, or combining ASQ results with other know risk factors such as intraventricular hemorrhage, periventricular leucomalacia, retinopathy and bronchopulmonary dysplasia might further improve these figures, and may help to identify early preterm born children who are at risk for NDI at a younger age [32].

The major strength of our study was using NDI as an indicator of developmental delay, not relying only on BSIDIII test results because several children failed on the neurological or sensory examination and not on BSIDIII, or had missing BSIDIII scores, due to their neurological or sensory impairment. Additionally, as far as we are aware our study was the first in its kind to compare ASQ3 with BSIDIII at original and adjusted cut-offs at 24 months’ corrected age.

The most important weakness of our study was that we did not administer the language scale of BSIDIII, thus hindering comparison with other studies. Adding the BSID III language scale might have improved specificity and positive predictive value of the ASQ3 as 25% of the children with ASQ failure without NDI failed on the ASQ communication domain (S5 Table). Another weakness was using U.S. cut-off scores for ASQ3 and BSIDIII, because Dutch cut-off scores were not yet available for these tools. But even so, using US cutoffs for both measures might reduce error. Finally, even with adjusted thresholds, only 7% of all children (born before 32 weeks of gestation) had NDI, thus limiting the power of our study. Our subgroup of children born before 28 weeks of gestation was relatively small (n = 61) and the rate of NDI was not much higher (8%); consequently ASQ3 performed not substantially better (S3 Table).

Our results suggest that the ASQ3 is a useful developmental screening tool to help detect NDI. Failure on ASQ3 requires to be followed by formal neurodevelopmental assessment including BSIDIII. This approach could have important implications. It reduces the workload and is more cost-effective since smaller groups of high-risk children are selected, who require in-depth assessment. Available resources could thus be transferred to benefit larger groups of high-risk children for whom neurodevelopmental testing might also be important, such as moderate and late preterm-born children, children post-asphyxia, children with congenital heart defects or other major congenital malformations requiring neonatal surgery.

Our findings might also encourage researchers to use this two-step approach to include, otherwise costly developmental outcome assessments in randomized controlled trials and cohort studies involving high-risk pregnant women and newborns. Finally, it might boost serial ASQ use in preventive child health care in community settings and low resource settings, thus fitting in with the policy of the American Academy of Pediatrics (AAP) to increase early detection of NDI and may help children with developmental delays to gain timely access to early intervention services at younger age [1]. These services may include integrated developmental support programs with physiotherapy, pre-logopedics and orthopedagogic support.

In Conclusion, this study demonstrated that ASQ3 is a simple, valid and cost-effective screening tool to help identify and exclude NDI in very preterm-born children at the corrected age of two years. It might be used to select those children who need more extensive neurodevelopmental testing, such as BSIDIII.

## Supporting Information

S1 Table Characteristics and outcomes of included vs excluded children. (PDF)

S2 Table Children with NDI and their neonatal- and neurodevelopmental characteristics. (PDF)

S3 Table Subgroup analyses of children ≤ 28 weeks and > 28 weeks of gestational age for ASQ3 versus NDI with original (<70) and adjusted BSID thresholds (<80 and <85), sensitivity, specificity, negative and positive predictive values. (PDF)

S4 Table Bayley scores in relation to ASQ outcomes. (PDF)

S5 Table ASQ3 failures and the domains for children with NDI or No NDI. (PDF)
