# Supplementary material for: The Ages and Stages Questionnaire and Neurodevelopmental Impairment in Two-Year-Old Preterm-Born Children
Source: PLoS One. 2015 Jul 20;10(7):e0133087. doi: 10.1371/journal.pone.0133087 (PMC4508030; doi:10.1371/journal.pone.0133087)
Supplement: S1 Table — (PDF) [file pone.0133087.s001.pdf]

S1 Table. Characteristics and outcomes of included vs excluded children.

Characteristic		All Eligible N=301	Included N=224	Excluded N=77	P*
**Gestational age**	≤ 28^{+0} wks	80 (27%)	61 (27%)	19 (25%)	0.661
	> 28^{+0} wks	221 (73%)	163 (73%)	58 (75%)	
**SGA[1]**	≤P10	66 (22%)	45 (20%)	21 (27%)	0.189
	>P10	235 (78%)	179 (80%)	56 (73%)	
**Gender**	Male	169 (56%)	122 (54%)	47 (61%)	0.316
	Female	132 (44%)	102 (46%)	30 (39%)	
**Multiple birth**	Multiple	99 (33%)	71 (32%)	28 (36%)	0.452
	Single	202 (67%)	153 (68%)	49 (64%)	
**Ethnicity mother**	Caucasian	274/298 (92%)	206/221 (93%)	68/77 (88%)	0.174
	Non-Caucasian	24/298 (8%)	15/221 (7%)	9/77 (12%)	
**Education mother[2]**	Low	40/275 (15%)	28/208 (13%)	12/67 (18%)	0.369
	Middle-High	235/275 (85%)	180/208 (87%)	55/67 (82%)	
**Necrotizing enterocolitis**	Yes	13 (4%)	9 (4%)	4 (5%)	0.661
	No	288 (96%)	215 (96%)	73 (95%)	
**IVH or PVL[3]**	Yes	15 (5%)	13 (6%)	2 (3%)	0.265
	No	286 (95%)	211 (94%)	75 (97%)	
**BPD[4]**	Yes	28 (9%)	24 (11%)	4 (5%)	0.150
	No	273 (91%)	200 (89%)	73 (95%)	
**Sepsis[5]**	Yes	8 (3%)	5 (2%)	3 (4%)	0.434
	No	293 (97%)	219 (98%)	74 (96%)	
**NDI**	Yes	10/271 (4%)	10 (4%)	0/47 (0%)	0.140
	No	261/271 (96%)	214 (96%)	47/47 (100%)	
	Unknown	30	0	30	
**ASQ3**	Failure	84/301 (28%)	61 (27%)	23/77 (30%)	0.656
	Pass	217/301 (72%)	163 (73%)	54/77 (70%)	
**BSIDIII**	C motor Score	102±12 (262)	102±13 (221)	102±11 (41)	1.00
	Cognitive Score	102±12 (262)	102±12 (221)	101±9 (41)	0.612
	Unknown (N)	39	3	36	

Data are presented as numbers (N/n), percentages (%), or mean +/- SD (n) of the BSID3 scores of all eligible children, included and excluded children. *P values are the results of comparisons between included and excluded children by Chi-square analyses, or student T-tests for BSIDIII scores. ASQ3: Ages and Stages Questionnaire, Third Edition, BSIDIII: Bayley Scales of Infant and Toddler Development, Third Edition.NDI: neurodevelopmental impairment: BSIDIII cognitive score or composite motor score of <70, bilateral blindness/deafness or cerebral palsy. Failure ASQ3: a score of >2 SD below the mean score for the US reference group on any domain.

[1] SGA: small for gestational age, birth weight < P10 on Dutch reference growth chart.

[2] low education of mother is less than 5 years high school education.

[3] IVH or PVL: intraventricular hemorrhage ≥ grade 3 (Papile) or periventricular leukomalacia ≥ grade 3. (De Vries)

[4] BPD: bronchopulmonary dysplasia defined as oxygen dependence at 36 weeks' gestation.

[5] Sepsis: clinical signs of septicemia and positive blood culture.
