# Supplementary material for: The Ages and Stages Questionnaire and Neurodevelopmental Impairment in Two-Year-Old Preterm-Born Children
Source: PLoS One. 2015 Jul 20;10(7):e0133087. doi: 10.1371/journal.pone.0133087 (PMC4508030; doi:10.1371/journal.pone.0133087)
Supplement: S2 Table — (PDF) [file pone.0133087.s002.pdf]

S2 Table: Children with NDI and their neonatal – and neurodevelopmental characteristics

child	sex	GA	BW	complications of prematurity	NBRS score	NDI reason	BSID CS	BSID CMS	GMFCS level	neurology	hearing	vision	ASQ failure domain
1	F	31+4	1875	None	1	CP	80	*	4	CP	normal	mild	GM, ProS
2	M	29+6	1365	Sepsis	2	CP	115	*	3	CP	normal	normal	GM
3	F	31+1	1780	IVH 1	4	CP	85	76	3	CP	normal	normal	GM, ProS
4	M	25+0	690	PDA, NEC3b-surgery, sepsis, meningitis, IVH1, BPD and ROP1	10	CP	*	*	2	CP	normal	normal	all 5 domains
5	M	25+0	630	PDA-surgery, sepsis, BPD, ROP1	8	blind	90	94	1	normal	normal	blind	Com, PerS
6	M	27+0	985	PDA-surgery, sepsis ROP1, ALGO refer	5	deaf	105	97	1	normal	normal	normal	Com, ProS
7	M	27+0	1165	PDA, sepsis, PVL3	6	BSID<70	55	46	4	CP	deaf	normal	all 5 domains
8	M	31+3	1970	none	0	BSID<70	55	61	1	mild	normal	normal	GM, Com ProS, PerS
9	M	29+1	1080	asphyxia, sepsis, PVL1	4	BSID<70	90	64	1	mild	normal	mild	GM, FM, Com, PerS
10	M	31+1	1630	PVL1	1	BSID<70	60	82	1	normal	normal	mild	GM, Com, ProS, PerS
11	F	28+5	970	PDA-surgery, PVL1 BPD, ROP1	7	BSID<80	75	76	1	normal	normal	mild	GM, FM, ProS
12	M	29+6	1465	IVH1	2	BSID<80	80	79	1	normal	normal	normal	FM
13	M	27+3	1130	None	2	BSID<85	80	88	1	normal	normal	normal	PerS
14	F	30+2	1080	PDA	1	BSID<85	90	82	1	normal	normal	normal	none
15	M	30+6	2005	None	2	BSID<85	80	100	1	normal	normal	normal	none

Children with neurodevelopmental impairment (NDI) based on neurological examination (CP), severe hearing or vision impairment or BSID III score < 70, or 80 or 85. Children 1-10 NDI with BSIDIII threshold <70. Children 1-12 at BSIDIII threshold <80, and children 1-15 at BSIDIII threshold <85. M male and F female. GA gestational age, BW birth weight. Complications of prematurity are asphyxia (Apgar score <5 at 5 min), sepsis/meningitis, persistent ductus arteriosis (PDA) plus surgery, necrotizing enterocolitis (NEC) plus Bell stage plus surgery, intraventricular hemorrhage (IVH) plus Papile grade, periventricular leucomalacia (PVL) plus De Vries grade, bronchopulmonary dysplasia (BPD), retinopathy of prematurity (ROP) plus grade, hearing (ALGO) refer, and none implies none of these above. NBRS Neurobiologic Risk Score, NDI is neurodevelopmental impairment , BSID Bayley Scales of Infant Development III, CS cognitive score, CMS composite motor score, * incomplete test due to CP. GMFCS Gross Motor Function Classification System. CP cerebral palsy on neurological examination. ASQ failure domains are GM Gross Motor, FM Fine Motor, Com communication, ProS Problem Solving and PerS Personal Social).
