# Supplementary material for: The Ages and Stages Questionnaire and Neurodevelopmental Impairment in Two-Year-Old Preterm-Born Children
Source: PLoS One. 2015 Jul 20;10(7):e0133087. doi: 10.1371/journal.pone.0133087 (PMC4508030; doi:10.1371/journal.pone.0133087)
Supplement: S3 Table — (PDF) [file pone.0133087.s003.pdf]

S3 Table. Subgroup analyses of children ≤ 28 weeks and > 28 weeks of gestational age for ASQ3 versus NDI with original (<70) and adjusted BSID thresholds (<80 and <85), sensitivity, specificity, negative and positive predictive values.

	**Subgroup ≤ 28 wks n = 61**	**Subgroup > 28 wks n = 163**	**Total group N = 224**
**NDI with BSIDIII<70**	4 (7%)	6 (4%)	10 (5%)
**NDI with BSIDIII<80**	4 (7%)	8 (4%)	12 (5%)
**NDI with BSIDIII<85**	5 (8%)	10 (6%)	15 (7%)
**ASQ3 failure**	25 (41%)	36 (22%)	61 (27%)
**Sensitivity***	5/5 (100%)	8/10 (80%)	13/15 (87%)
**Specificity**	36/56 (64%)	125/153 (82%)	161/209 (77%)
**NPV**	36/36 (100%)	125/127 (98%)	161/163 (99%)
**PPV**	5/25 (20%)	8/36 (22%)	13/61 (21%)

Data are presented as numbers and percentages. ASQ3: Ages and Stages Questionnaire, Third edition. Failure ASQ3: a score of >-2 SD below the mean score for the U.S. reference group on any domain BSIDIII: Bayley Scales of Infant and Toddler Development, Third Edition. NDI with BSIDIII<70: neurodevelopmental impairment with BSIDIII cognitive score or composite motor score of <70, bilateral blindness/deafness and/or cerebral palsy. NDI with BSIDIII<80: neurodevelopmental impairment with BSIDIII cognitive score or composite motor score of <80, bilateral blindness/deafness and/or cerebral palsy. NDI with BSIDIII< 85: neurodevelopmental impairment with BSIDIII cognitive score or composite motor score of <85, bilateral blindness/deafness and/or cerebral palsy. *Sensitivity, specificity, negative predictive values (NPV) and positive predictive values (PPV) are calculated for ASQ3 vs NDI with BSIDIII with threshold <85.
