# Supplementary material for: The Ages and Stages Questionnaire and Neurodevelopmental Impairment in Two-Year-Old Preterm-Born Children
Source: PLoS One. 2015 Jul 20;10(7):e0133087. doi: 10.1371/journal.pone.0133087 (PMC4508030; doi:10.1371/journal.pone.0133087)
Supplement: S4 Table — (PDF) [file pone.0133087.s004.pdf]

S4 Table. Bayley scores in relation to ASQ outcomes.

Group children	Cognitive Score mean ± SD	C Motor Score Mean ± SD
**All included children** N=224	102±12	102±13
**All who passed ASQ3** n=163	105±11	105±11
**All who failed ASQ3** n=61	95±13	95±14
**Failure ASQ3 and NDI** n=10	82±22	74±18
**Failure ASQ3 but no NDI** n=51	98±10	98±11

Data are presented as means ± SD of the BSID3 cognitive scores and composite motor score. ASQ3: Ages and Stages Questionnaire, Third Edition, BSIDIII: Bayley Scales of Infant and Toddler Development, Third Edition. NDI: neurodevelopmental impairment: BSIDIII cognitive score or composite motor score of <70, bilateral blindness/deafness or cerebral palsy. Failure ASQ3: a score of >2 SD below the mean score for the US reference group on any domain. Children who passed the ASQ3 had significantly higher cognitive scores and composite motor scores than children who failed the ASQ3 (P<0.001), and to those who failed the ASQ without NDI (P<0.001) using the independent sample T-test.
