# Supplementary material for: The Ages and Stages Questionnaire and Neurodevelopmental Impairment in Two-Year-Old Preterm-Born Children
Source: PLoS One. 2015 Jul 20;10(7):e0133087. doi: 10.1371/journal.pone.0133087 (PMC4508030; doi:10.1371/journal.pone.0133087)
Supplement: S5 Table — (PDF) [file pone.0133087.s005.pdf]

S5 Table. ASQ3 failures and the domains for children with NDI or No NDI.

	Total N=224	NDI with BSIDIII < 85 n=15	No NDI BSIDIII ≥ 85 n=209	No NDI and Failure ASQ3 n=48
**Failure ASQ3**	61 (27%)	13 (87%)	48 (23%)	48 (100%)
**Domain ASQ3**	Failure n (%)	Failure n (%)	Failure n (%)	Failure n (%)
**Communication**	18 (8%)	6 (40%)	12 (6%)	12 (25%)
**Gross Motor**	34 (15%)	9 (60%)	25 (12%)	25 (52%)
**Fine Motor**	12 (5%)	5 (33%)	7 (3%)	7 (15%)
**Problem Solving**	14 (6%)	7 (47%)	7 (3%)	7 (15%)
**Personal Social**	18 (8%)	7 (47%)	11 (5%)	11 (23%))

Data are presented as numbers and percentages.
ASQ3: Ages and Stages Questionnaire, Third edition.
Failure ASQ3: a score of >2 SD below the mean score for the U.S. reference group on any domain
BSIDIII: Bayley Scales of Infant and Toddler Development, Third Edition.
NDI with BSIDIII<85: neurodevelopmental impairment with BSIDIII cognitive score or composite motor score of <85, bilateral blindness/deafness and/or cerebral palsy.
